# Proposal for the Use of an Industrial Membrane System for Lactose Recovery From Whey: Adaptation of Technology Used in Protein Concentration

**DOI:** 10.1111/1750-3841.71155

**Published:** 2026-06-21

**Authors:** Danieli Bucior, Gabriela Mesquita Bruel, Ilizandra Aparecida Fernandes, Clarice Steffens, Eunice Valduga, Juliana Steffens

**Affiliations:** ^1^ Department of Food Engineering Regional Integrated University of the Upper Uruguay and the Missions Erechim Brazil

## Abstract

This study evaluated the feasibility of recovering and concentrating lactose from ultrafiltration (UF) permeate of sweet whey using nanofiltration (NF) and reverse osmosis (RO) under industrial conditions. The objective was to adapt existing whey protein recovery systems to valorize the lactose fraction. UF permeates from different batches were concentrated by NF at 16–25 bar, with 16 bar combined with diafiltration showing the best performance for lactose concentration. The NF concentrate was further concentrated by RO, increasing lactose content and reducing mineral levels. Spray drying produced a powder containing 94.10% lactose, with low moisture (0.98%), low water activity (0.258), and moderate hygroscopicity (5.20%). FTIR and physicochemical analyses confirmed lactose purity, and the overall process yield reached 87.68%. The integration of NF, RO, and drying proved technically efficient for lactose valorization and demonstrated that membrane systems traditionally used for protein recovery can be adapted for industrial lactose production, contributing to the added value of whey. A complete mass balance and environmental assessment would further strengthen the evaluation of its economic and environmental performance.

**Practical Applications:**

This research demonstrates the feasibility of converting cheese whey, a commonly discarded dairy byproduct, into high‐purity lactose using industrial membrane technologies. The proposed process achieved good yield and operational efficiency by adapting existing systems already used in the dairy industry. This approach supports the valorization of whey, reducing environmental impact while generating a high‐value ingredient. The recovered lactose has wide applicability, including use in the food industry, pharmaceuticals, cosmetics, and potential bioenergy applications. Overall, the study contributes to improving sustainability and value generation within the dairy production chain.

## 1 Introduction

The growing demand for safe, nutritious, and sustainable foods has driven innovations in the dairy industry. Among the main areas of innovation is the valorization of byproducts such as whey, generated primarily during cheese production. In 2024, global whey production reached 16 million tons, with moderate growth expected in the coming years. Brazil stands out in this scenario, with an estimated production of 300 thousand metric tons in 2025 (Indexbox 2024).

Whey is a nutrient‐rich matrix, with lactose as one of its main constituents, and holds high potential for applications in the food, pharmaceutical, cosmetic, and biotechnological industries. The valorization of this disaccharide has become strategic in light of its growing international demand, where its average price reached USD 1165 per metric ton in 2025 (Global Dairy Trade 2025). However, the efficient recovery of lactose faces technical challenges, particularly after ultrafiltration (UF), due to its low residual content and the presence of salts, which hinder purification and crystallization.

In this context, membrane separation technologies such as nanofiltration (NF) and reverse osmosis (RO) have been widely applied to concentrate lactose and selectively remove salts (Fancello et al. 2024). The integration of these processes with spray drying enables the production of powdered lactose with high purity, low moisture content, good solubility, and stability characteristics desired for various industrial applications (Kaur et al. 2020; Tsermoula et al. 2023).

The adoption of these technologies has been led by industries in countries such as France, Denmark, New Zealand, and the United States, which transform whey into higher value‐added products, such as protein concentrates and functional ingredients. In Brazil, industries in the southern region have emerged as pioneers in applying UF, NF, and RO systems for the concentration and standardization of whey (Cuartas‐Uribe et al. 2009). However, the use of UF permeate, which can account for up to 13 kg per 1 kg of protein isolate, remains limited (Ramírez‐Navas et al. 2018).

The use of membranes for whey protein concentration/isolation is already well established at the industrial level and is considered an efficient, standardized, and economically viable process (Ostertag et al. 2023). Nonetheless, applying the same membrane systems for lactose concentration is still underexplored, as many studies report the use of NF systems specifically designed for lactose concentration, often employing spiral‐wound polyamide membranes with larger membrane surface areas and configurations optimized for carbohydrate fractionation and concentration. In contrast, the approach adopted in this study does not rely on dedicated lactose‐processing equipment, since the novelty of this work lies precisely in adapting an existing protein recovery system to perform the sequence UF → NF → RO using the same membrane platform, thereby enabling lactose concentration within an already installed membrane infrastructure. This gap highlights the need for studies assessing the feasibility of adapting conventional industrial protein recovery systems to obtain concentrated lactose.

Among the main technical barriers to lactose recovery are membrane fouling and scaling, resulting from salt precipitation, such as calcium phosphate, which compromise process efficiency and require frequent cleaning (Halfwerk et al. 2023). The success of the process depends on parameters such as membrane selectivity, chemical resistance, and hydrophobicity, as well as maintaining adequate temperatures to prevent the degradation of sensitive components while complying with current legislation (Brazil Ministry of Agriculture, Livestock, and Supply 2018).

Thus, technologies that combine high technical performance with environmental sustainability are essential for whey valorization. The recovery of lactose through membrane separation and its conversion into powder by spray drying represents a viable alternative for reducing losses, adding value to the byproduct, and generating new functional ingredients. In this context, the aim of this study was to apply membrane systems traditionally used for industrial protein recovery to lactose separation. To this end, an integrated process of NF and RO membrane separation and spray drying of the concentrate was evaluated, focusing on its technical feasibility, process efficiency, final product quality, and environmental sustainability.

## 2 Materials and Methods

### 2.1 Sample Collection and Preparation

**FIGURE 1:**
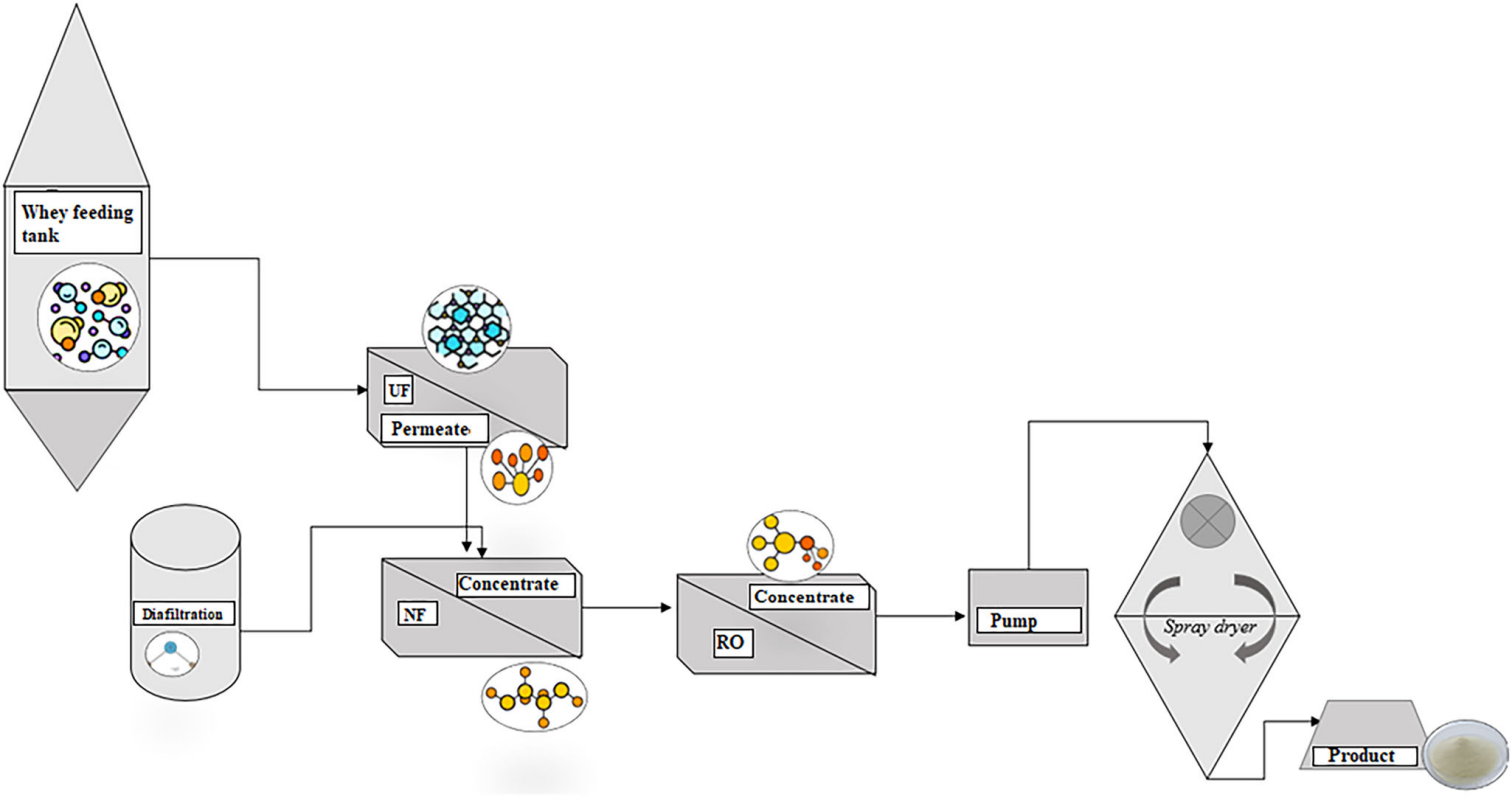
Simplified schematic representation of lactose concentration and dehydration.

Figure 1 presents a simplified schematic of the steps involved in the recovery and dehydration of lactose in an industrial system. The tests were carried out in an industrial system. Permeates from whey UF were collected from nine different batches of a dairy industry located in northern Rio Grande do Sul, Brazil, which receives and concentrates whey from several cheese plants distributed across the southern region of the country. After characterization of the nine batches and statistical analysis of the results, low variation was observed (CV < 3.0%). Therefore, the nine permeate batches were grouped into three representative sets based on similarity in physicochemical composition. Each group consisted of three original UF batches and was used as an independent experimental replicate for the next concentration step by NF.

In continuous operation mode, the permeates passed through an NF membrane with a molecular weight cutoff (MWCO) of 200 Da (∼150 Da) and an average pore diameter of 0.7 nm. The NF unit was connected to a tank supplied with potable water and equipped with recirculation, enabling diafiltration (DF). The NF/DF concentrate was then subjected to RO using a membrane with an MWCO of <200 Da (∼100 Da) and an average pore diameter of <0.5 nm. The system was connected through a plate heat exchanger for temperature adjustments. Subsequently, the RO concentrate was dried in a spray dryer.

Samples (*n* = 3) were collected from the different UF batches and at the NF, NF/DF, and RO concentration stages. They were stored under refrigeration (10°C) and physicochemically characterized in terms of lactose, protein, fat, total minerals and mineral components (sodium, calcium, and potassium), total acidity, pH, and total solids. The dehydrated sample was also characterized.

### 2.2 Membrane System Configuration and Operating Conditions

The UF system is equipped with polysulfone membranes with a nominal MWCO of 500 kDa, arranged in spiral configuration in series, with a total operational area of 30 m^2^, operating at 4 bar and 10°C. This UF step corresponds to the industrial whey protein concentration process, where proteins are retained in the retentate fraction, while lactose, minerals, and other low‐molecular‐weight compounds pass to the permeate.

Although the nominal MWCO is relatively high compared with the molecular weight of individual whey proteins, effective protein retention occurs under industrial operating conditions due to protein aggregation, protein–mineral interactions, and the formation of a concentration polarization layer and fouling deposits on the membrane surface, which reduce the effective pore size during operation.

The UF permeates were concentrated in an industrial system using NF membranes (DOW‐NF90‐2540), with an MWCO of approximately 200 Da, according to the manufacturer's specifications, composed of flat‐surface polyamide with a multilayer structure and internal meshes that provide mechanical support and enhance flow. Tests were performed at three different pressures (16, 20, and 25 bar) at 10°C, combined with DF and recirculation, to evaluate the effects of operational conditions on permeate flux and lactose concentration. DF was performed in continuous mode with water recirculation, in which potable water was added to the feed tank, while permeate was simultaneously removed, maintaining the system under recirculation until the desired concentration factor (CF) was reached. The temperature of 10°C was maintained throughout the process to preserve the stability of sensitive whey components, such as lactose and proteins, and to comply with current legislation. According to RIISPOA and Normative Instruction No. 76/2018 from the Ministry of Agriculture, Livestock, and Supply (Brazil Ministry of Agriculture, Livestock, and Supply 2018), whey must be maintained at 10°C or lower from its collection to processing to prevent microbial growth and alterations in pH and acidity. This condition ensures both technological quality and legal compliance of the process. Samples were characterized physicochemically.

The selected operating pressures of 16, 20, and 25 bar fall within the operational range for NF processes, enabling the study of the impact of transmembrane pressure on yield, selectivity, and permeate flux. Higher pressures generally increase permeate flux but may also intensify fouling and reduce selective separation efficiency (Habert et al. 2006).

Based on the condition that yielded the highest lactose concentration in the NF‐treated permeate (16 bar), the system was combined with DF with water recirculation. The DF step was performed in constant‐volume mode, in which potable water was continuously added to the feed tank at the same rate as permeate removal, maintaining the retentate volume approximately constant throughout the process. A CF of 1.3 was obtained during NF. This value was defined a priori as the target operational condition based on the industrial process used in the dairy plant. The NF process was stopped when this CF was reached, corresponding to an average processing time of approximately 120 min. The processed volume reached a CF of 1.3; that is, the final retentate volume corresponded to approximately 77% of the initial volume. The NF/DF concentrate was physicochemically characterized.

The mineral separation observed during NF can also be interpreted considering electrostatic interactions between ions and the membrane surface. NF membranes such as DOW‐NF90 typically exhibit a negatively charged surface at the pH range of whey (approximately 6.0–6.2). Under these conditions, ion separation is strongly influenced by the Donnan exclusion mechanism, which promotes the rejection of multivalent ions due to electrostatic repulsion and charge balance effects. Consequently, divalent anions such as sulfate and phosphate tend to be strongly rejected, while divalent cations such as calcium may also present higher retention due to co‐ion exclusion and interactions with counter‐ions in solution. Furthermore, the increase in mineral retention observed with increasing transmembrane pressure (Table 2) may be associated with enhanced concentration polarization at the membrane surface, which modifies the local ionic environment and reinforces electrostatic exclusion effects (Van der Bruggen and Vandecasteele 2003).

The NF/DF concentrate (16 bar, 10°C) was further processed for lactose recovery in an industrial system using an RO membrane (thin‐film composite polyamide, SW30HRLE), which, according to the manufacturer's specifications, presents an MWCO lower than 200 Da (∼100 Da). The RO system was operated under the same temperature conditions as NF/DF. The RO system was operated at 16 bar, corresponding to the standard operating pressure currently used in the industrial plant for whey concentration and protein separation. In this study, the same industrial module and operating conditions were maintained to evaluate the feasibility of lactose recovery without modifying the existing process configuration.

To evaluate the performance of the membrane separation system (NF at 16, 20, and 25 bar, and RO at 16 bar), the permeate fluxes of ultrapure water and whey were assessed. Permeates were collected every 5 min for 2 h, and the volumetric permeate flux was calculated using Equation (1):

(1) $$J_{p}=\frac{V}{A.t}$$

where *J*_p_ is the permeate flux (L/m^2^·h); *A* is the effective membrane area (317 m^2^); *V* is the collected permeate volume (L); and *t* is the permeation time (h).

In the UF, NF, and RO steps, the process was stopped when a CF of approximately 1.3 was reached. This target was defined a priori based on the standard industrial process conditions employed at the dairy plant to ensure consistency with existing protein recovery protocols. The total duration of each representative concentration run was approximately 15 h, or until the target CF was achieved. Membrane cleaning was performed either at the end of the full process or after 15 h of continuous operation to mitigate fouling.

After each membrane separation step, membranes were cleaned, beginning with rinsing with potable water. Subsequently, alkaline detergent (ENER F5 MB, Enerquímica) was circulated, with the outlet pH maintained between 6.0 and 9.0 throughout the operation. The cleaning cycle lasted approximately 30 min, followed by rinsing with potable water to remove detergent residues, according to the cleaning protocol adopted by the industry.

### 2.3 Dehydration of Lactose Concentrates

The lactose concentrate obtained by RO was dried using a spray dryer (LabMaq, model YC–5.0), equipped with a 0.5‐mm nozzle, maximum feed pump flow of 5 L/h, drying chamber temperature of 102°C, 6000 W power, and atomization pressure of 3 bar. The dehydrated product samples were characterized in terms of yield, lactose content, water activity, moisture, acidity, pH, structural groups, solubility, and hygroscopicity. The low solubility observed for the dehydrated product (Table 4) may be related to the rapid water evaporation during spray drying, which can lead to the formation of amorphous lactose. This state is metastable and highly hygroscopic, which can undergo subsequent crystallization and affect the overall dissolution profile of the powder (Shrestha et al. 2007).

### 2.4 Analytical Determinations

#### 2.4.1 Lactose

Lactose was determined by high‐performance liquid chromatography (HPLC, Shimadzu LC‐20 AD) with a refractive index detector. The column used was Rezex RHM‐Monosaccharide H+ (300 × 7.80 mm). The column oven temperature was 50°C. The flow rate was 1.0 mL/min, with a 20‐µL injection volume.

The mobile phase was prepared by adding 440 µL of sulfuric acid to a 1‐L volumetric flask and completing the volume with distilled water. The solution was filtered through a 0.45‐µm membrane filter. For analysis, 1.2 g of sample from the membrane separation system was weighed and dissolved in 50 mL of ultrapure water at 50°C, diluted to 100 mL, and homogenized. Then, 10 mL of this solution was further diluted in 90 mL of ultrapure water and homogenized. The solution was filtered through a 0.45‐µm membrane.

The standard solution was prepared by weighing 1.0 g of lactose standard and diluting to 100 mL with the mobile phase. Then, 10 mL of this solution was transferred to a 100‐mL volumetric flask and diluted with the mobile phase. The solution was homogenized and filtered through a 0.45‐µm membrane.

The lactose concentration (%) was quantified using Equation (2):

(2) $$X\left(\%\right)=\frac{A_{t}\times m_{s}\times C\times 100}{A_{s}\times m_{t}}$$

where *A*_t_ and *A*_s_ are the peak areas of sample and standard solutions, respectively; *m*_s_ is the standard mass (g); *m*_t_ is the sample mass (g); and *C* is the purified lactose standard.

#### 2.4.2 Total Solids

The total solids content of the samples was determined by the gravimetric method in a recirculation oven (New Lab—NL 80–252) at 88°C (dehydrated product) and 105°C (liquid) for approximately 4 h and/or until constant weight. Seashore sand was used to facilitate heat transfer.

#### 2.4.3 Total Protein

Total nitrogen content was determined by the Kjeldahl method, according to AOAC Method No. 920.123 (AOAC International 2005), using a Kjeldahl digestion and distillation system (Hanon—SH220F). The protein content was calculated using a nitrogen‐to‐protein conversion factor of 6.38.

#### 2.4.4 Total Acidity

Total acidity was determined by titration with N/9 sodium hydroxide (Dornic solution) (AOAC International 2005). Results were expressed as grams of lactic acid per 100 g of product.

#### 2.4.5 pH

The pH was measured by the potentiometric method, according to AOAC Method No. 4022 (AOAC International 2005), using a pH meter (Quimis—Q400AS), with readings taken directly from the sample.

#### 2.4.6 Total Minerals

Total mineral content (ash) was determined by incineration in a muffle furnace (Lavoisier Furnace, SSFM 6.7 L) at 555°C and quantified gravimetrically.

#### 2.4.7 Mineral Components

Calcium, sodium, and potassium contents were analyzed by flame atomic absorption spectrometry (DIGIMED—DM‐63), calibrated with standard sodium, potassium, and calcium solutions (1–100 ppm).

#### 2.4.8 Total Fat

Total fat content was determined using a butyrometer according to Normative Instruction No. 30 (Brazil Ministry of Agriculture, Livestock, and Supply 2020).

#### 2.4.9 Solubility

Solubility was determined using 7.5 g of dehydrated sample dissolved in 100 mL of distilled water under agitation (9000 rpm, 5 min).

#### 2.4.10 Hygroscopicity

For hygroscopicity analysis, dehydrated samples (2 g) were weighed into 100 × 20 mm glass plates and placed in a desiccator containing distilled water for 16 h at 20°C (Cavalcante et al. 2018). Results were quantified according to Equation (3):

(3) $$\mathrm{Hygroscopicity}\;\left(\%\right)=\frac{M_{F}-M_{I}}{m}\times 100$$

where *M*_F_ is the final mass (g), *M*_I_ is the initial mass (g), and *m* is the sample mass (g).

#### 2.4.11 Water Activity (*a*_w_)

The water activity of the dehydrated samples was determined directly using a water activity analyzer (Novasina, Labtouch).

#### 2.4.12 Functional Groups

Functional groups of the dehydrated sample were determined by Fourier transform infrared (FTIR) spectroscopy (Agilent, Cary 630 ZnSe PC Bundle) equipped with an attenuated total reflection (Diamond ATR) accessory. Surface spectra were collected in transmittance mode in the range of 650–4000 cm^−1^, with a resolution of 2 cm^−1^.

#### 2.4.13 Overall Yield

The overall yield of the dehydrated sample was calculated according to Equation (4):

(4) $$\mathrm{Yield}\;\left(\%\right)=\frac{m_{F}}{m_{I}}\times 100$$

where *m*_F_ is the final lactose mass (g) and *m*_I_ is the initial lactose mass (g).

### 2.5 Statistical Treatment

The results (*n* = 3) were statistically analyzed by analysis of variance (ANOVA) and comparison of means using Tukey's test, with the aid of Statistica software version 5.0 (Statsoft Inc., USA) at a 95% confidence level.

A two‐way ANOVA was performed to examine the effects of NF pressure and permeate batches, as well as their interaction, on the variables total solids, lactose, pH, acidity, and mineral components (sodium, potassium, and calcium). Means were compared using Tukey's test (*p* < 0.05), with the aid of Past software version 4.32.

Pearson correlation analysis and principal component analysis (PCA) were performed with the aid of XLSTAT 2020, Free version.

## 3 Results and Discussion

### 3.1 Permeate Fluxes and Membrane Separation System

**FIGURE 2:**
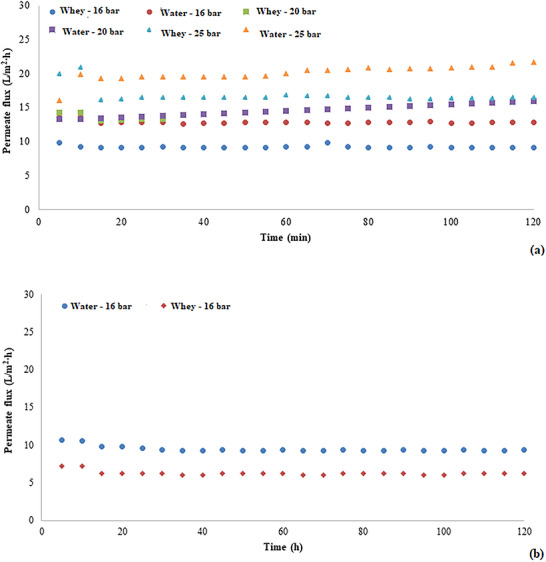
Permeate fluxes of water and whey from NF at 16, 20, and 25 bar (a) and RO at 16 bar (b).

Figure 2 presents the permeate fluxes of water and whey as a function of permeation time through NF membranes (16, 20, and 25 bar) and RO membranes (16 bar). The permeate flux curves presented in Figure 2 correspond to specific experimental tests conducted under the same industrial conditions as the full‐scale runs. These measurements were monitored for an initial characterization phase of 120 min to assess flux stability and decline patterns. In Figure 2a, it can be observed that the permeate fluxes obtained with water remained practically constant over time, regardless of the applied pressure. This behavior was expected due to the absence of solutes capable of inducing concentration polarization or significant fouling (Cheryan 1998). In contrast, the trials conducted with whey showed the typical initial decline in flux, attributed to the formation of a polarization layer by proteins, salts, and lactose, followed by a quasi‐steady‐state regime (Baker 2004; Cassano et al. 2017). This initial flux decline observed is primarily attributed to concentration polarization, where solutes accumulate at the membrane–solution interface, creating a boundary layer that increases osmotic pressure and reduces the effective driving force (Baker 2004). Increasing the operating pressure (16, 20, and 25 bar) resulted in a proportional rise in flux for both water and whey. However, in the case of whey, the relative gain was lower, since the additional resistance imposed by solutes limits the expected linear response according to Darcy's law (Habert et al. 2006).

In Figure 2b, referring to RO at 16 bar, the behavior was analogous: water exhibited constant flux, while whey presented an initial decline followed by stabilization, as also observed in NF. Nevertheless, the fluxes obtained in RO were lower than those in NF, a direct reflection of the higher structural density and selectivity of the RO membrane, which increases hydraulic resistance to transport (Baker 2004). Moreover, the higher solute rejection intensifies the osmotic effect at the membrane interface, further reducing the effective flux. It should also be noted that the RO experiments were conducted at a transmembrane pressure of 16 bar, which corresponds to the pressure currently used in the industrial plant. At the total solids concentration observed in the NF concentrate (Table 2), mainly composed of lactose and mineral salts, the osmotic pressure of the solution may approach the applied pressure, thereby reducing the effective driving force for permeation. This effect likely contributed to the relatively low permeate flux observed during the RO step.

A direct comparison between the membranes operating with whey at 16 bar shows that NF yielded significantly higher fluxes than RO under the same pressure conditions. This difference can be explained by the higher intrinsic permeability of the NF membrane and the lower contribution of apparent osmotic pressure (Cassano et al. 2017; Habert et al. 2006). From a practical standpoint, these results indicate that for applications where volumetric productivity is a priority, NF represents a more advantageous alternative. On the other hand, RO, even operating with lower fluxes, ensures higher solute rejection and may be more suitable for processes requiring high selectivity and greater retentate concentration (Baker 2004; Cheryan 1998).

### 3.2 Physicochemical Characteristics of the UF Permeate and the NF Concentrate

Table 1 presents the physicochemical characteristics of the permeate obtained by UF of different whey batches. As expected, lactose constituted the main component of the permeate, with values ranging from 86.84 to 91.25 g/100 g_d.b._, with no significant differences among batches (*p* > 0.05). This result confirms the high passage of lactose through the UF membrane, due to its low molecular weight, a feature already reported in similar studies (Li et al. 2024).

**TABLE 1:** Physicochemical characteristics of the ultrafiltration (UF) permeate from different whey batches.

Parameters	Batches									CV (%)
	1	2	3	4	5	6	7	8	9	
Lactose (g/100 g_d.b._)	90.00^a^ (0.20)	91.25^a^ (0.23)	88.67^a^ (0.47)	86.84^a^ (0.19)	90.95^a^ (0.09)	86.84^a^ (0.19)	90.70^a^ (0.01)	90.86^a^ (0.03)	89.60^a^ (0.01)	2.12
Fat (g/100 g)	0.0833^ab^ (0.02)	0.1001^a^ (0.02)	0.0367^bc^ (0.02)	0.0034^c^ (0.06)	0.0233^c^ (0.02)	0.0034^c^ (0.06)	0.0233^c^ (0.02)	0.0034^c^ (0.06)	0.0233^c^ (0.02)	55.32
Protein (g/100 g_d.b._)	2.11^d^ (0.01)	2.15^b^ (0.01)	2.10^d^ (0.01)	2.15^bc^ (0.01)	2.23^a^ (0.01)	2.15^b^ (0.01)	2.23^a^ (0.02)	2.15^b^ (0.01)	2.12^cd^ (0.02)	0.54
Solids (g/100 g)	15.24^bc^ (0.09)	16.23^a^ (0.58)	15.01^bc^ (0.74)	15.74^ab^ (0.19)	15.55^ab^ (0.09)	15.74^ab^ (0.19)	14.57^c^ (0.01)	15.22^bc^ (0.03)	15.91^ab^ (0.02)	2.14
pH	6.10^b^ (0.01)	6.32^a^ (0.06)	6.30^a^ (0.00)	6.27^a^ (0.02)	6.31^a^ (0.07)	6.27^a^ (0.02)	6.31^a^ (0.07)	6.26^a^ (0.01)	6.33^a^ (0.03)	0.61
Acidity (° Dornic)	41.18^a^ (0.10)	35.03^e^ (0.06)	40.40^b^ (0.05)	40.35^bc^ (0.05)	40.10^d^ (0.10)	40.35^bc^ (0.05)	40.10^cd^ (0.07)	40.50^b^ (0.00)	40.10^d^ (0.10)	0.16
Minerals (g/100 g)	0.55^de^ (0.01)	0.70^a^ (0.01)	0.63^b^ (0.01)	0.56^d^ (0.00)	0.40^g^ (0.02)	0.51^f^ (0.01)	0.60^c^ (0.02)	0.63^b^ (0.01)	0.54^e^ (0.02)	0.82
Sodium (mg/100 g)	365.33^e^ (0.58)	436.3^c^ (0.60)	364.66^e^ (0.58)	364.83^e^ (0.29)	633.3^a^ (0.60)	364.83^e^ (0.58)	633.3^b^ (0.58)	364.33^e^ (0.58)	426.33^d^ (0.58)	0.13
Potassium (mg/100 g)	1767^c^ (0.64)	1355.33^e^ (0.58)	1425.33^d^ (0.73)	1320.66^f^ (0.58)	2091.66^a^ (1.15)	1425.33^d^ (0.58)	1956.33^b^ (0.58)	1125.33^g^ (0.70)	2090.63^a^ (0.55)	0.07
Calcium (mg/100 g)	77.33^c^ (0.58)	51.66^c^ (0.58)	59.00^d^ (0.73)	60.00^d^ (0.65)	102.67^a^ (0.58)	90.48^b^ (0.14)	60.25^d^ (0.17)	60.21^d^ (1.06)	90.37^b^ (0.18)	0.94

*Note*: Means (standard deviation) followed by the same letters/lines indicate no significant difference (*p* > 0.05, Tukey's test).

Abbreviation: d.b., dry basis.

Protein content remained close to 2.1 g/100 g_d.b._, demonstrating that whey protein retention was effective, as expected for UF membranes, which act as a selective barrier for macromolecules (Bronstein and Monte Alegre 1998). The lipid fraction showed low concentration and greater relative variation (CV 55.32%), reflecting the low permeability of fat, but also possible differences in the initial whey composition depending on the raw material used.

Total solids remained between 14.57 and 16.23 g/100 g, values consistent with those reported for UF permeates and within the 12%–30% range described by Li et al. (2024) for concentrated whey. The stability of total solids is strongly associated with the high contribution of lactose in the composition, which confers homogeneity among batches.

Regarding pH, values ranged from 6.10 to 6.33, all within the range established by legislation (Brazil Ministry of Agriculture, Livestock, and Supply 2020) for sweet whey (6.0–6.8). Titratable acidity showed slight variation among batches (35.03 to 41.18 °D) but remained consistent with the expected classification, reinforcing that the evaluated permeate did not undergo significant spontaneous fermentation (Pereira et al. 2017). This stability in pH and acidity is essential, as these parameters directly influence mineral solubility, protein stability, and fouling formation during membrane separation.

With respect to minerals, significant variability among batches was observed, mainly for sodium, potassium, and calcium. Sodium content ranged from 364.33 to 633.3 mg/100 g, while potassium showed even more pronounced differences (1125.33–2091.66 mg/100 g). This fluctuation is related to the type of cheese produced and the technological process employed, since the addition of salts during production directly impacts the electrolyte profile of the permeate. Calcium ranged from 51.66 to 102.67 mg/100 g, evidencing the influence of raw material and possible differences in membrane retention efficiency under different operating conditions. The rejection of ions during the NF process can be explained by the Donnan equilibrium model, which describes the electrostatic interaction between charged solutes and the fixed charges on the membrane surface. This mechanism, combined with size exclusion, accounts for the high retention of multivalent ions compared to monovalent ones (Vandezande et al. 2008).

Overall, the results indicate that UF permeate presents a stable composition in terms of lactose, protein, and pH, but may exhibit significant variability in mineral content depending on the characteristics of the source whey. This heterogeneity must be considered in process standardization, as it affects both the final quality of the permeate and its use in different technological or nutritional applications.

**FIGURE 3:**
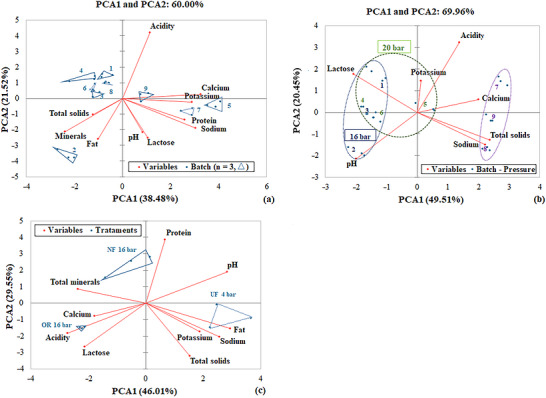
Principal component analysis of the physicochemical characteristics of the ultrafiltration (UF) permeate at 4 bar from different whey batches (a) and of the nanofiltration (NF) concentrated fractions at 16, 20, and 25 bar from different ultrafiltration (UF) whey permeate batches (b), as well as the comparative concentrated fractions from the ultrafiltration (UF) system at 4 bar, nanofiltration (NF) at 16 bar, and reverse osmosis (RO) at 16 bar.

Figure 3 presents the PCA of the physicochemical characteristics of the permeates from different whey batches processed by UF. Variables are represented as vectors, and the longer the vector, the better it explains the variability among variables. The first (PC1) and second (PC2) dimensions explained 60% of the total variance, with PC1 accounting for 38.48% and PC2 for 21.52%. It was observed that there was discrimination among the whey permeate batches analyzed: Batch 2 stood out due to its composition regarding fat, solids, and minerals, while Batches 5 and 7 showed higher concentrations of calcium, potassium, sodium, and protein. Batches 1, 3, 4, 6, 8, and 9 belonged to a larger group closer to the acidity variable, indicating higher values.

In Table 1, it can be observed that pH ranged from 6.10 to 6.33, while titratable acidity varied between 35.03 and 41.18 °D. This negative correlation is confirmed in Figure 3, where the vectors of pH and acidity are oriented in opposite directions, indicating that batches with higher acidity tend to have lower pH values. In addition, minerals such as calcium, potassium, and sodium tend to concentrate similarly across different batches, evidencing a positive correlation among them.

Figure 3a also highlights the relationship between lactose and pH, suggesting that batches with higher lactose concentrations tend to exhibit higher pH values. This trend can be observed in Table 1, where Batch 2, which presented one of the highest lactose concentrations (91.25 g/100 g_d.b._), also exhibited one of the highest pH values (6.32).

The relationship observed between higher lactose concentrations and higher pH values in the UF permeate is consistent with the literature. Studies have shown that lactose does not directly contribute to medium acidification; however, when metabolized by lactic acid bacteria, it is converted into lactic acid, thereby promoting a reduction in pH (Pereira et al. 2017). Thus, batches with higher lactose contents tend to present higher pH values, as observed in Batch 2 of this study (91.25 g/100 g_d.b._; pH 6.32), suggesting low fermentation or absence of acidulants. Moreover, the permeate composition also influences chemical equilibrium. According to Cuartas‐Uribe et al. (2009), the high mineral content, such as calcium phosphate, may affect pH and system stability, especially after concentration. Studies have also identified the presence of organic acids (hydroxypyruvic acid, gluconic acid, malonic acid, and ribonic acid) in whey permeate (Tsermoula et al. 2023), which can alter acidity and should be considered when interpreting pH. The combination of these factors, such as lactose content, fermentation, mineral composition, and presence of secondary organic acids in the permeate, may influence system buffering, contributing to pH stability.

Table 2 presents the physicochemical characteristics of the NF‐concentrated fractions at pressures of 16, 20, and 25 bar, obtained from different UF whey permeate batches. It can be observed that the solids content increased with applied pressure, ranging from 11.97%–13.45% at NF 16 bar to 16.13%–17.44% at NF 25 bar. This result was expected due to the greater water removal. Although lactose remained high under all conditions, it exhibited a slight reduction as pressure increased, decreasing from approximately 89%–91% at NF 16 bar to 85%–87% at NF 25 bar, which may be related to the retention of other solid components that reduce the relative lactose fraction.

**TABLE 2:** Physicochemical characteristics of nanofiltration (NF) concentrated fractions at pressures of 16, 20, and 25 bar from different ultrafiltration (UF) whey permeate batches.

Treatment	Batch	Characteristics						
		Solids (g/100 g)	Lactose (g/100 g)	pH	Acidity (°D)	Sodium (mg/100 g)	Potassium (mg/100 g)	Calcium (mg/100 g)
NF 16 bar	01	11.97^f^ (0.06)	91.37^a^ (0.93)	6.06^f^ (0.01)	42.07^c^ (0.06)	266.67^g^ (2.08)	1320.67^b^ (1.15)	82.67^e^ (0.58)
	02	13.45^c^ (0.05)	88.89^a^ (0.96)	6.21^a^ (0.01)	37.00^f^ (0.001)	323.00^e^ (3.00)	1110.50^b^ (5.13)	55.50^h^ (0.58)
	03	12.30^d^ (0.02)	89.43^a^ (0.50)	6.20^ab^ (0.01)	40.90^e^ (0.001)	289.00^f^ (2.08)	1229.00^b^ (3.51)	63.00^f^ (2.08)
	LI^*^	12.57^b^ (0.68)	89.93^a^ (1.29)	6.15^a^ (0.07)	39.99^c^ (2.29)	293.11^c^ (24.61)	1219.00^b^ (23.57)	67.22^c^ (12.18)
NF 20 bar	04	12.25^d^ (0.04)	90.16^a^ (1.01)	6.16^cd^ (0.02)	41.00^d^ (0.06)	265.00^g^ (0.58)	1093.00^b^ (1.00)	63.00^fg^ (0.15)
	05	12.42^e^ (0.02)	89.08^a^ (1.08)	6.15c (0.02)	41.07^d^ (0.12)	421.67^d^ (1.53)	1316.67^b^ (3.06)	150.20^a^ (1.59)
	06	12.39^de^ (0.12)	89.01^a^ (1.33)	6.19^bd^ (0.01)	40.90^de^ (0.001)	289.67^f^ (2.08)	1228.67^b^ (3.51)	63.67^g^ (2.08)
	LII^*^	12.35^c^ (0.11)	89.30^a^ (1.07)	6.17^a^ (0.02)	40.98^b^ (0.09)	326.5^b^ (74.35)	1212.78^b^ (64.78)	92.30^b^ (43.44)
NF 25 bar	07	16.13^b^ (0.15)	87.50^b^ (0.66)	5.91^g^ (0.01)	44.00^a^ (0.001)	474.67^b^ (0.58)	1981.67^a^ (4.51)	111.67^c^ (0.58)
	08	17.35^a^ (0.06)	87.05^b^ (0.32)	6.10^e^ (0.01)	40.00^e^ (0.001)	658.00^a^ (1.53)	2003.00^a^ (1.00)	105.00^d^ (2.52)
	09	17.44^a^ (0.03)	85.96^c^ (0.49)	6.07^f^ (0.01)	42.03^b^ (0.06)	425.33^c^ (2.52)	1982.00^a^ (6.08)	123.33^b^ (0.58)
	LIII^*^	16.97^a^ (0.64)	86.92^b^ (0.72)	6.03^b^ (0.09)	42.01^a^ (1.73)	519.22^a^ (106.02)	1988.88^a^ (11.25)	113.44^a^ (8.02)

*Note*: Means (standard deviation) followed by the same lowercase letters within columns indicate no significant difference (*p* > 0.05, Tukey's test) among batches. Data were statistically analyzed by two‐way ANOVA.

^*^ LI—batches at 16 bar pressure (*n* = 9); LII—batches at 20 bar pressure (*n* = 9); LIII—batches at 20 bar pressure (*n* = 9).

The pH ranged between 5.91 and 6.21, being lower in samples with higher solids concentration, while acidity showed a slight increase with pressure, reaching 44 °D at NF 25 bar, indicating greater retention of acidic compounds.

Sodium, potassium, and calcium levels increased progressively with pressure. The pressure averages (Table 2, LI, LII, and LIII) confirmed this trend, showing an effect of NF on component concentration, that is, with increasing pressure, greater concentration of solids and minerals occurred, while lactose on a dry basis decreased. In addition, pH tended to decrease and acidity increased, indicating greater retention of acidic compounds.

Two‐way ANOVA (Table S1) was performed to evaluate the effects of NF pressures (16, 20, and 25 bar) and different UF whey permeate batches on the physicochemical characteristics of the concentrated fractions. The results show that all variables analyzed were significantly affected (*p* < 0.05) by both pressure and batches, in addition to statistically significant interactions between these factors, except for lactose and potassium.

For total solids, the effects of pressure, batches, and their interaction were significant (*p* < 0.001), with pressure exerting the greatest impact (*F* = 1.03 × 10^4^). This result confirms that solid concentration increases with separation pressure. In the case of lactose, pressure had a significant effect (*p* < 0.001), while batches also influenced the results, but to a lesser extent (*p* = 0.007). The interaction was not statistically significant (*p* = 0.4349), indicating that the effects of pressure on lactose were similar across the evaluated batches.

The pH was affected by both pressure and batches (*p* < 0.001), as well as by the interaction between these factors, indicating that variations in acidity and the retention of compounds depend both on the applied pressure and on the characteristics of the permeate batches used. Acidity showed the highest *F*‐values in Table S1, reflecting a strong influence of pressure (*F* = 3550), batches (*F* = 7965), and their interaction (*F* = 2410), all with *p* < 0.001. This result demonstrates that the acidity of the concentrate strongly depends on the initial composition of the permeate and on the concentration effect promoted by NF. The ANOVA results indicate that NF significantly affects the physicochemical composition of the concentrates and that this influence is enhanced by increasing pressure.

Figure 3b shows the relationship between different physicochemical variables and sample batches subjected to different NF concentrate pressures (16, 20, and 25 bar). The PC1 axis explains 49.51% of the data variability, while the PC2 axis explains 20.45%, accounting for a total of 69.96% of the variance explained by the first two principal components. The variables analyzed include lactose, pH, acidity, potassium, calcium, sodium, and total solids. Each of these variables is represented by vectors, indicating their direction and influence in the principal component space. The samples from different batches are represented as points in the figure, according to their identification and pressure condition.

The distribution of batches in Figure 3b shows that the different pressures influenced the physicochemical composition of the samples. The batches at 16 bar are located closer to the lactose and pH variables, which is consistent with the values reported in Table 2, where these batches showed the highest lactose contents (89.93 g/100 g_d.b._) and pH values (6.15). Conversely, the batches concentrated at 25 bar are closer to the total solids, sodium, and calcium variables, confirming the higher values recorded in Table 2 for these parameters, with 16.97 g/100 g of total solids, 519.22 mg/100 g of sodium, and 113.44 mg/100 g of calcium, indicating a positive correlation among these variables.

The inverse relationship between pH and acidity, evidenced in Table S1, can also be observed in the PCA plot, where pH lies on the opposite side of acidity, reinforcing that increasing acidity is associated with decreasing pH as pressure increases. Furthermore, lactose shows higher values in batches processed at 16 and 20 bar, and its position indicates that increasing pressure reduces its concentration, likely due to greater retention of other components such as minerals and proteins. The behavior of total solids, calcium, and sodium, which increase with pressure, is also confirmed by PCA, associated with batches concentrated at 25 bar.

PCA indicates that the PC1 axis, representing 49.51% of data variability, separates batches based on total solids, sodium, and calcium on one side, and lactose and pH on the other, demonstrating the influence of pressure on these physicochemical parameters. The PC2 axis, representing 20.45% of variability, further explains batch distribution.

Therefore, the NF operating pressure significantly affects the composition of the concentrated fractions, promoting greater retention of solids and minerals at higher pressures, while lower pressures favor higher lactose content and elevated pH.

There is a strong negative correlation (Table S2) between total solids and lactose (*r* = –0.827), indicating that as solid concentration increases, lactose content decreases. This occurs because increasing NF pressure leads to greater retention of minerals and other components, reducing the proportion of lactose in the concentrated fraction. A significant positive correlation was also observed between total solids and sodium (*r* = 0.818), showing that sodium content increases as solids concentration rises, indicating greater retention of this mineral by the NF membrane.

The relationship between calcium and sodium (*r* = 0.612) demonstrates that samples with higher sodium content also tend to show higher calcium concentration, meaning that these minerals are retained in a similar manner during filtration. The inverse relationship between pH and acidity (*r* = –0.750) indicates that higher acidity corresponds to lower pH values.

Table 3 presents a comparison of the physicochemical characteristics of concentrated fractions obtained by UF at 4 bar, NF at 16 bar, and RO at 16 bar. Lactose content was highest in the RO concentrate (93.32 g/100 g_d.b._), significantly higher than in UF and NF, which did not differ from each other (∼89.93–89.97 g/100 g_d.b_.). This indicates that RO retained more lactose compared with NF and UF, possibly due to its lower porosity and greater rejection of low‐molecular‐weight compounds. Fat content was quantified only in UF (0.10 g/100 g), while it was removed in both NF and RO. This result is expected, as whey undergoes a skimming process before being subjected to NF and RO systems.

**TABLE 3:** Physicochemical characteristics of the concentrated fractions from ultrafiltration (UF), nanofiltration (NF), and reverse osmosis (RO).

System	Physicochemical characteristics									
	Solids (g/100 g)	Lactose (g/100 g_d.b._)	Fat (g/100 g)	Protein (g/100 g_d.b._)	pH	Acidity (°D)	Minerals (g/100 g)	Sodium (mg/100 g)	Potassium (mg/100 g)	Calcium (mg/100 g)
UF (4 bar)	15.99^a^ (0.86)	89.93^b^ (1.29)	0.10^a^ (0.03)	2.12^b^ (0.03)	6.24^a^ (0.12)	38.51^b^ (2.02)	0.63^b^ (0.07)	388.77^a^ (41.17)	1515.89^a^ (220.27)	60.66^a^ (14.43)
NF/DF (16 bar)	12.58^b^ (0.76)	89.97^b^ (1.24)	0.00^b^ (0.00)	2.31^a^ (0.03)	6.15^a^ (0.081)	39.99^b^ (2.65)	0.93^ab^ (0.20)	293.11^b^ (28.32)	1218.78^a^ (106.51)	67.23^a^ (14.01)
RO (16 bar)	15.15^a^ (0.032)	93.32^a^ (0.16)	0.00^b^ (0.00)	2.00^c^ (0.01)	5.91^b^ (0.012)	46.33^a^ (1.53)	1.02^a^ (0.01)	325.66^ab^ (0.57)	1235.00^a^ (1.67)	70.40^a^ (5.00)
CV (%)	4.55	1.14	—	1.09	1.39	5.96	14.59	8.59	10.68	18.14

*Note*: Means (*n* = 3, standard deviation) followed by the same lowercase letters/columns indicate no significant difference (*p* > 0.05, Tukey's test).

Abbreviation: d.b., dry basis.

Protein levels in UF, NF, and RO concentrates showed significant differences (*p* < 0.05). The higher protein proportion observed in the NF concentrate is mainly associated with the concentration effect caused by water removal and partial demineralization during NF, which increases the protein‐to‐total solids ratio when expressed on a dry basis, rather than indicating a preferential enrichment of proteins relative to the initial feed. Acidity also differed (*p* < 0.05) among the separation systems, being highest in RO (46.33 °D) and lowest in UF (38.51 °D). This demonstrates that RO promotes greater retention of acidic compounds. Therefore, RO not only removes target compounds but also alters the chemical characteristics of the concentrated solution, such as pH and acidity, due to the removal of buffering ions during the filtration process (Palmer and Kaminski 2022). Moreover, RO allows for greater retention of mineral salts, since its membrane has lower porosity and higher density, preventing ion passage into the permeate.

The result for the solids content shows a positive Pearson correlation with sodium (*r* = 0.886) and fat (*r* = 0.622). However, for solids and protein, there is a negative correlation (*r* = −0.708), and similarly between acidity and pH (*r* = −0.884). There is also a positive correlation between lactose and acidity (*r* = 0.700), suggesting that fractions richer in lactose tend to be more acidic. Additionally, mineral content shows a negative correlation with fat (*r* = −0.783). It should be noted that this correlation refers to the comparison among the UF, NF, and RO fractions obtained during the membrane concentration process. In contrast, the relationship discussed for the UF permeate batches in Table 1 reflects variability in the initial permeate composition prior to membrane concentration, and therefore, the two observations correspond to different datasets and process conditions.

Figure 3c shows the PC1 and PC2 axes explaining 75.57% of the data variability. The 4‐bar UF represents that the concentrated fraction has the highest contents of total solids, fat, sodium, and potassium. The 16‐bar NF is closer to the total protein, while the 16‐bar RO is more related to lactose, acidity, calcium, and minerals. This indicates that RO provides a higher concentration of lactose and minerals, while UF retains more solids and fat.

**FIGURE 4:**
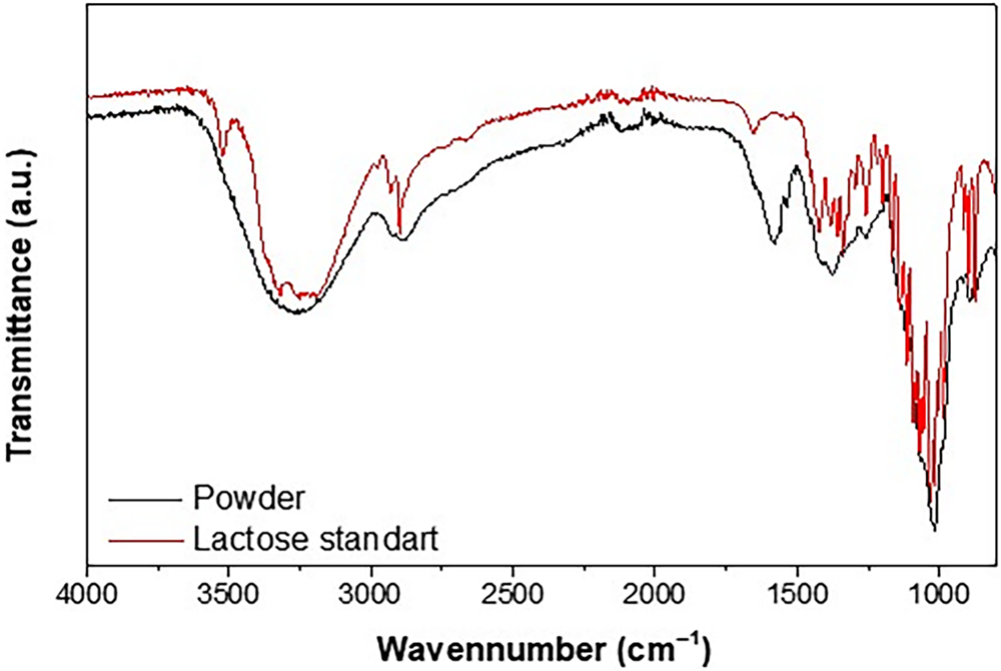
Analysis by Fourier transform infrared (FTIR) spectroscopy of lactose standard sample and lactose obtained from the RO concentrate (powder) at 16 bar and 10°C, with 94.10% lactose.

Figure 4 shows the FTIR spectrum bands associated with the functional groups of lactose and the spray‐dried concentrate obtained from RO (16 bar at 10°C), with 94.10% lactose (Table 4). In the spectrum (powder), 10 characteristic bands of lactose are observed at wavelengths 3254, 2883.1, 1884.1, 1772.3, 1017.6, 1114.5, 769.7, 874.1, 892.2, and 915 cm^−1^, compatible with hydroxyl stretching, aliphatic C─H bonds, and glycosidic bonds, typical of carbohydrates. In addition to these, eight other bands are observed that are not directly attributed to lactose, indicating the possible presence of residual proteins, fat, mineral salts (calcium, sodium, and potassium), or traces of organic compounds in low concentrations (Table 4).

**TABLE 4:** Physicochemical characteristics of the dehydrated lactose concentrate fraction obtained by spray drying.

Characteristics	Values
Lactose (%, d.b.)	94.10 ± 0.12
Proteins (%, d.b.)	1.30 ± 0.72
Fat (%, d.b.)	0.01 ± 0.001
Total minerals (%, d.b.)	2.56 ± 0.03
Sodium (mg/100 g)	404 ± 2.03
Calcium (mg/100 g)	72.00 ± 1.10
Potassium (mg/100 g)	1140 ± 0.78
Hygroscopic tendency (%)	5.20 ± 0.16
Solubility (%)	0.10 ± 0.01
Moisture (%)	0.98 ± 0.02
Water activity	0.258 ± 0.02
Yield (%)	87.68 ± 0.03

*Note*: Data represented as mean (*n* = 3) ± standard deviation.

Abbreviation: d.b., dry basis.

Lactose is a disaccharide composed of glucose and galactose joined by a β‐1,4‐glycosidic bond. In the FTIR spectrum of lactose standard, different absorption bands indicate the presence of characteristic functional groups, such as hydroxyls, which are responsible for the hygroscopic properties of lactose (range 3200–3600 cm^−1^). Aliphatic stretches are nonaromatic organic compounds, meaning they do not have benzene rings. In general, they are linear or branched carbon chains, which can be saturated or unsaturated, which is characteristic of carbohydrates. Carbonyls, associated with oxidized lactose derivatives, are present in the range between 2800 and 3000 cm^−1^. It also presents glycosidic bonds (range 1000–1200 cm^−1^), essential for the structure of lactose, including the glycosidic bond between glucose and galactose (range between 900 and 1200 cm^−1^) (Corrêa 2017).

Although FTIR does not allow for the direct quantification of mineral salts such as sodium (Na^+^), potassium (K^+^), and calcium (Ca^2+^), their presence is demonstrated in the spectrum. When compared to the spectrum of a standard lactose sample (Figure 4a), the analyzed RO lactose concentrate presents a greater number of secondary bands, which suggests lower purity.

However, for the RO lactose concentrate to meet pharmaceutical‐grade requirements, additional purification steps are necessary to increase the purity level (>99%) due to the presence of a protein fraction, fat, and mineral salts (Table 4). Even small amounts of water can mask or alter the characteristic spectral bands of the molecule (Conceição et al. 2019). Nevertheless, the dehydrated lactose concentrate is suitable for food applications with low moisture content (0.98%), associated with low water activity (0.258), which makes the product more stable from microbiological, agglomeration, and biochemical oxidation reaction standpoints (Duarte 2024). The relatively low solubility observed for the spray‐dried powder (Table 4) may be associated with the complex composition of the material, which includes lactose together with residual proteins, lipids, and mineral salts rather than pure lactose. In addition, structural modifications occurring during spray drying, such as partial lactose crystallization and protein–mineral interactions, may reduce powder dispersion in water and consequently decrease the apparent solubility.

In the differentiation between crystalline forms, lactose can exist as α‐lactose monohydrate and anhydrous β‐lactose, based on absorption bands and differences in infrared absorption patterns (Montes 2004; Soares and Petrovick 1999). Although FTIR provides information about the molecular structure of lactose, it does not allow an unequivocal identification of the specific crystalline polymorph present in the spray‐dried powder. The lactose in the product may therefore include partially amorphous regions commonly formed during spray drying, which can influence properties such as hygroscopicity and water activity.

According to De Zaldivar Ribeiro (2021), the FTIR spectrum of lactose presents characteristic bands that can be associated with the main functional groups present in its molecular structure. For the spectra found in the powder sample, the band around 3254 cm^−1^ corresponds to the stretching vibration of the hydroxyl group (─OH), which appears as a broad band between 3200 and 3600 cm^−1^, typical of compounds with a high capacity to form hydrogen bonds, common in sugars. The band located at 2883.1 cm^−1^ is related to the stretching of C─H bonds from aliphatic groups, also indicating the presence of interactions via hydrogen bonding, common in carbohydrates. The bands observed at 1884.1 and 1772.3 cm^−1^ may be associated with the vibrations of C─H bonds present in the glucose and galactose rings that constitute lactose. Eventually, these bands may also indicate the presence of carbonyl groups (C═O) if there are any oxidized derivatives of lactose, since the region between 1600 and 1800 cm^−1^ is typical for this vibration.

The bands located at 1017.6 and 1114.5 cm^−1^ are attributed to the vibration of ether bonds (C─O─C), corresponding to the glycosidic bonds that link the glucose and galactose units. Finally, the bands observed in the regions of 769.7, 874.1, 892.2, and 915 cm^−1^ indicate the presence of C─OH‐type vibrations and are directly associated with the glycosidic bond between the monosaccharides. These are characteristic of vibrations present between 900 and 1200 cm^−1^ and are particularly useful for distinguishing different structural forms of lactose. This thus allows for confirming the presence of this disaccharide, in addition to identifying possible structural variations or interactions with other components in the dehydrated concentrated whey matrix.

The hygroscopic tendency of 5.20% (Table 4) demonstrates a moderate capacity to absorb moisture from the environment, which may be related to the presence of hydroxyl groups that interact with water. Therefore, packaging with a moisture barrier is required to preserve the product's stability (Silva 2014).

In general, the process of concentrating and recovering lactose from whey using membrane separation technologies followed by dehydration demonstrates an efficient strategy for valorizing this dairy industry co‐product. The application of steps such as UF, NF/DF, and RO allows for the selective separation of components, retaining proteins and allowing lactose and mineral salts to pass into the permeate. This is then concentrated by spray drying, resulting in a powder with a high lactose content (94.10%) and low levels of moisture and water activity, which are ideal characteristics for stability and storage.

The process mass balance demonstrates good solids recovery (a yield of 87.68%) with a significant reduction in organic load and effective utilization of the soluble whey fraction. Thus, the integrated use of membranes and thermal drying not only contributes to the dairy plant's sustainability but also generates an ingredient with potential application in food and pharmaceutical formulations, promoting the complete utilization of a byproduct previously considered waste.

## 4 Conclusion

This study, carried out at an industrial scale, demonstrated that the integrated application of NF, RO, and spray drying technologies is a viable and efficient alternative for the recovery and concentration of lactose from the UF permeate of sweet whey. The optimized operating condition (NF at 16 bar combined with DF) enabled the selective removal of salts and acids, favoring the production of a concentrate further enriched in lactose by RO. The final product, with 94.10% purity, low moisture, and low water activity, exhibited suitable physicochemical properties to ensure stability, solubility, and potential for industrial application.

With an overall yield of 87.68%, the process proved to be technically effective, economically feasible, and environmentally sustainable. Conducting this study directly in an industrial environment reinforces its practical applicability and technological transfer potential, making it a realistic solution for the valorization of an abundant byproduct of the dairy industry that is often discarded.

Therefore, adapting membrane systems already established for protein recovery represents a promising strategy for the integral utilization of whey, contributing to the valorization of dairy processing streams. However, a complete mass balance including DF water consumption and the composition of NF and RO permeate streams would be necessary to fully assess the environmental and economic performance of the proposed process.

## Author Contributions

**Danieli Bucior**: investigation, conceptualization, methodology, validation, formal analysis, writing – original draft. **Gabriela Mesquita Bruel**: conceptualization, methodology, visualization. **Ilizandra Aparecida Fernandes**: investigation, formal analysis, visualization. **Clarice Steffens**: formal analysis, visualization. **Eunice Valduga**: writing – review and editing, writing – original draft, formal analysis, supervision, data curation, visualization. **Juliana Steffens**: data curation, supervision, writing – review and editing, visualization.

## Conflicts of Interest

The authors declare no conflicts of interest.

## Supporting information




**Table S1**: jfds71155‐sup‐0001‐TableS1.docx

**Table S2**: jfds71155‐sup‐0001‐TableS1.docx

**Table S3**: jfds71155‐sup‐0003‐TableS3.docx
