# Supplementary material for: Proposal for the Use of an Industrial Membrane System for Lactose Recovery From Whey: Adaptation of Technology Used in Protein Concentration
Source: J Food Sci. 2026 Jun 21;91(6):e71155. doi: 10.1111/1750-3841.71155 (PMC13284522; doi:10.1111/1750-3841.71155)
Supplement: Supplementary file 1 — Table S1: jfds71155‐sup‐0001‐TableS1.docx [file JFDS-91-0-s003.docx]

**Table S1** Two-way ANOVA of the physicochemical characteristics of nanofiltration (NF) concentrated fractions at pressures of 16, 20, and 25 bar from different ultrafiltration (UF) whey permeate batches.

| **Variables** | **Sources of variation** | **Sum of Squares** | | **DF** | **Medium square** | **F** | ***P* -value** |
| --- | --- | --- | --- | --- | --- | --- | --- |
| **Total solids** | Pressure | 122.19 | 2 | | 61.09 | 1.03 10^4 | < 0.001* |
|  | Batch | 4.21 | 2 | | 2.10 | 355.50 | < 0.001* |
|  | Interaction | 2.66 | 4 | | 0.66 | 112.60 | < 0.001* |
| **Lactose** | Pressure | 45.37 | 2 | | 22.69 | 30.25 | < 0.001* |
|  | Batch | 10.03 | 2 | | 5.02 | 6.69 | 0.007* |
|  | Interaction | 2.99 | 4 | | 0.747 | 0.996 | 0.4349^ns^ |
| **pH** | Pressure | 0.108 | 2 | | 0.054 | 357.2 | < 0.001* |
|  | Batch | 0.071 | 2 | | 0.035 | 234.2 | < 0.001* |
|  | Interaction | 0.035 | 4 | | 0.009 | 57.68 | < 0.001* |
| **Acidity** | Pressure | 18.40 | 2 | | 9.20 | 3550 | < 0.001* |
|  | Batch | 41.29 | 2 | | 20.65 | 7965 | < 0.001* |
|  | Interaction | 24.99 | 4 | | 6.25 | 2410 | < 0.001* |
| **Sodium** | Pressure | 268,096 | 2 | | 134,048 | 3.58 10^4 | < 0.001* |
|  | Batch | 106,491 | 2 | | 53,245.5 | 1.42.10^4 | < 0.001* |
|  | Interaction | 32,428.4 | 4 | | 8,107.09 | 2167 | < 0.001* |
| **Potassium** | Pressure | 6.24 10^6 | 2 | | 3.12 10^6 | 1.05 | 0.0371^*^ |
|  | Batch | 4.99 10^6 | 2 | | 2.49 10^6 | 0.837 | 0.449^ns^ |
|  | Interaction | 8.71 10^6 | 4 | | 2.18 10^6 | 0.731 | 0.583^ns^ |
| **Calcium** | Pressure | 9,637.43 | 2 | | 4,818.71 | 2297 | < 0.001* |
|  | Batch | 2,177.06 | 2 | | 1,088.53 | 519 | < 0.001* |
|  | Interaction | 14,587.20 | 4 | | 3,646.79 | 1739 | < 0.001* |

DF: degrees of freedom; *significant difference (p<0.05); ns: not significant.
