# Supplementary material for: Proposal for the Use of an Industrial Membrane System for Lactose Recovery From Whey: Adaptation of Technology Used in Protein Concentration
Source: J Food Sci. 2026 Jun 21;91(6):e71155. doi: 10.1111/1750-3841.71155 (PMC13284522; doi:10.1111/1750-3841.71155)
Supplement: Supplementary file 2 — Table S2: jfds71155‐sup‐0001‐TableS1.docx [file JFDS-91-0-s001.docx]

**Table S2** Pearson correlation matrix (r) of the physicochemical characteristics of nanofiltration (NF) concentrated fractions at 16, 20, and 25 bar pressures from different ultrafiltration (UF) whey permeate batches.

| **Variables** | **Solids** | **Lactose** | **pH** | **Acidity** | **Sodium** | **Potassium** | **Calcium** |
| --- | --- | --- | --- | --- | --- | --- | --- |
| **Solids** | **1** |  |  |  |  |  |  |
| **Lactose** | **-0.827** | **1** |  |  |  |  |  |
| **pH** | -0.555 | 0.318 | **1** |  |  |  |  |
| **Acidity** | 0.198 | -0.141 | **-0.750** | **1** |  |  |  |
| **Sodium** | **0.818** | **-0.665** | -0.416 | 0.095 | **1** |  |  |
| **Potassium** | 0.029 | 0.075 | -0.081 | 0.111 | 0.011 | **1** |  |
| **Calcium** | 0.463 | -0.427 | -0.481 | 0.452 | **0.612** | 0.022 | **1** |

*Values in bold differ significantly at the 95% confidence level (r > 0.61).
