# Supplementary material for: Proposal for the Use of an Industrial Membrane System for Lactose Recovery From Whey: Adaptation of Technology Used in Protein Concentration
Source: J Food Sci. 2026 Jun 21;91(6):e71155. doi: 10.1111/1750-3841.71155 (PMC13284522; doi:10.1111/1750-3841.71155)
Supplement: Supplementary file 3 — Table S3: jfds71155‐sup‐0003‐TableS3.docx [file JFDS-91-0-s002.docx]

**Table S3** Pearson correlation matrix (r) of the physicochemical characteristics of the concentrated fractions from ultrafiltration (UF), nanofiltration (NF), and reverse osmosis (RO) membranes.

| **Variables** | **Lactose** | **Fat** | **Solids** | **Protein** | **Minerals** | **Calcium** | **Sodium** | **Potassium** | **pH** | **Acidity** |
| --- | --- | --- | --- | --- | --- | --- | --- | --- | --- | --- |
| **Lactose** | **1** |  |  |  |  |  |  |  |  |  |
| **Fat** | -0.312 | **1** |  |  |  |  |  |  |  |  |
| **Solids** | 0.242 | **0.622** | **1** |  |  |  |  |  |  |  |
| **Protein** | **-0.681** | -0.109 | **-0.708** | **1** |  |  |  |  |  |  |
| **Minerals** | 0.383 | **-0.783** | -0.263 | -0.029 | **1** |  |  |  |  |  |
| **Calcium** | 0.426 | -0.239 | -0.424 | -0.119 | -0.097 | **1** |  |  |  |  |
| **Sodium** | -0.083 | **0.836** | **0.886** | -0.327 | -0.419 | -0.574 | **1** |  |  |  |
| **Potassium** | -0.254 | **0.788** | 0.321 | -0.193 | **-0.850** | 0.274 | 0.395 | **1** |  |  |
| **pH** | **-0.837** | 0.544 | 0.092 | 0.567 | -0.476 | **-0.681** | 0.469 | 0.189 | **1** |  |
| **Acidity** | **0.700** | -0.588 | -0.074 | **-0.636** | 0.404 | 0.529 | -0.501 | -0.189 | **-0.884** | **1** |

*Values in bold differ significantly at the 95% confidence level (r > 0.61).
